# Virological investigation of fatal rabies in a minor bitten by a mongrel in Nigeria

**DOI:** 10.11604/pamj.2021.39.129.24218

**Published:** 2021-06-15

**Authors:** Ishaya Sini Tekki, Bernard Anyebe Onoja, Adedayo Omotayo Faneye, Ismaila Shittu, Georgina Ndejika Odaibo, David Olufemi Olaleye

**Affiliations:** 1 National Veterinary Research Institute, PMB 01, Vom, Nigeria,; 2 Department of Virology, College of Medicine, University of Ibadan, Ibadan, Nigeria,; 3 University College Hospital, Ibadan, Nigeria

**Keywords:** Human rabies, classical rabies virus, rural community, direct fluorescent antibody test, reverse transcription polymerase chain reaction, Nigeria

## Abstract

Rabies is a deadly viral disease transmitted through bites of infected animals. Outbreaks continue to escalate in Africa, with fatalities in humans, especially in rural areas, but are rarely reported. About 40% casualties occur among children of < 15 years. A 5-year-old boy on referral from a Primary Health Care Centre to a tertiary hospital presented with anxiety, confusion, agitation, hydrophobia, photo-phobia and aero-phobia, seven weeks after he was bitten by a stray dog in a rural community in Nigeria. The patient did not receive post-exposure prophylaxis and died 48 hours post admission. Confirmatory diagnosis was rabies and the phylogenetic analysis of the partial N-gene sequence of the virus localized it to Africa 2 (genotype 1) Lyssaviruses. There was 95.7-100% and 94.9-99.5% identity between the isolate and other genotype 1 Lyssaviruses and 100% homology with rabies viruses from Mali, Burkina Faso, Senegal and Central African Republic.

## Introduction

Rabies is a zoonotic disease caused by rabies virus (RABV), in the genus *Lyssavirus* and family *Rhabdoviridae*. RABV genome has a 50-nucleotide leader sequence followed by five genes that encode for 5 proteins namely: nucleoprotein (N), phosphoprotein (P), matrix protein (M), glycoprotein (G) and polymerase (L) [1]. Rabies is mostly transmitted to humans through bites of infected animals, especially dogs, cats and bats [2]. An annual global estimate of 59,000 human mortality attributed to rabies through dog bite occur mostly in developing countries of Asia and Africa, representing a daily death of about 160 persons. Africa alone accounts for 36.4% of these deaths [3], occurring mostly in rural communities where population of unvaccinated dogs are high [4]. Generally, children of less than 15 years of age are the highest casualties because of their inquisitiveness and lack of restraint in teasing or playing with dogs [5]. Rabies poses a bigger problem in rural communities of sub-Saharan Africa [6] where costs of vaccinating dogs and Post Exposure Prophylaxis for exposed humans are not easily affordable [7]. Although a notifiable disease, it is under-reported due to ignorance, poverty and lack of effective surveillance system [3]; also by limited diagnostic facilities and inadequate political will from the government. In Nigeria, previous studies focused on the ecology [8], past records of dog and human rabies [9] and diagnosis of animal rabies [10]. Despite the high case-fatality rate, little attention is being given to laboratory confirmation of rabies in people with illnesses presenting with symptoms that incriminate rabies, in Nigeria. In this study, we describe laboratory investigation of suspected rabies in a 5-year-old boy who presented with hydrophobia, photo-phobia and aero-phobia in a tertiary health institution in south-western Nigeria.

## Methods

**Study design:** saliva and cerebrospinal fluid (CSF) specimen were collected from a 5-year old boy presenting with anxiety, confusion, agitation, hydrophobia, photo-phobia and aero-phobia. He was referred from a Primary Health Care Centre to the University College Hospital (UCH) Ibadan, Nigeria in 2014. He was reportedly bitten by a local dog seven weeks earlier in a rural community. No human post-exposure prophylaxis (PEP) was administered and there was no information about vaccination status of the dog.

**Mice inoculation:** thirty micro-litres of 10% (v/v) suspension each, of saliva and CSF was inoculated intra-cerebrally into suckling mice housed with the dame. They were observed for 28 days for signs of physical disorientation such as ruffled coat, in-coordination, tremors, paralysis and death. Ethical approval was obtained from institutional animal use and care committee (AUCC) of National Veterinary Research Institute Vom, Nigeria (NVRI AUCC REF No: AEC/02/23/15) in accordance with the EU Directive 2010/63/EU for animal experiments.

**Direct fluorescent antibody test:** DFA was performed on impression smears of mice brain tissue from day 5 and above post-inoculation (PI), using Flourescein-Isothiocynate (FITC) labelled rabies antibody (anti-nucleoprotein antibody -N4-15) (Onderstepoort Veterinary Institute, Pretoria, South Africa) according to manufacturer´s instructions; employing the methods described by Dean *et al*. [11]. Brain smears of mice infected with Challenge Virus Strain of rabies virus (CVS) were used as positive control while smears of brain of uninfected mouse served as negative control.

**RNA extraction and RT-PCR detection of N gene:** total ribonucleic acid (RNA) was extracted from tissues of infected mice brain using RNA purification kit (Jena Biosciences®, Germany). Amplification of partial N gene of RABV by reverse transcription polymerase chain reaction (RT-PCR) assay was performed using primers described by Heaton *et al*. [12]. For complementary deoxyribonucleic acid (cDNA) synthesis, 1μL of JW12 (10μm) forward primer and 5μL RNA were incubated at 94°C for one minute and immediately cooled on ice for 5 minutes. GeneAmp Gold RNA PCR reagent kit (Applied Biosystems, USA) was used to prepare a 14 μL RT reaction mixture containing 2.5 μL nuclease free water, 4.5 μL 5x PCR buffer, 2.2 μL dNTPs (10mM each), 2.5 μL DTT (100 mM), 1.5 μL MgCl_2_ (25mM), 0.4 μL RNase inhibitor (20U/μL), 0.4 μL Reverse transcriptase enzyme, followed by addition of 6 μL cooled primer-RNA mixture. The 20 μL volume mixture was then incubated at 42°C for 90 minutes.

A hemi nested (hn) PCR as previously described by Heaton *et al*. [12] with modification, was used to amplify the N gene of RABV in a 25μL reaction mixture containing 5.0 μL of 5X PCR buffer, 1.5 μL MgCl_2_ (25mM), 1.0 μL dNTPs mix (10mM each), 1.0 μL of each primers (10μM) JW12, JW6 (DPL, M), 0.5μL Rnase Inhibitor (20U/μL), 0.5μL AmpliTaq Enzyme, 4.0 μL cDNA and 10.5 μL nuclease-free PCR grade water in the first round PCR. In the second round PCR, a mix of 5.0 μL of 5X PCR buffer, 1.5 μL MgCl_2_ (25mM), 1.0 μL dNTPs mix (10mM each), 1.0 μL (10μM) of each primers JW12, JW10 (DLE2, ME1 and P), 0.5μL Rnase Inhibitor (20U/μL), 0.5μL AmpliTaq Enzyme, 1.0 μL of first round product and 12.5 μL nuclease-free PCR grade water to make up the final volume of 25 μL reaction. Amplification of N gene was performed on GeneAmp 9700 thermal cycler (Applied Biosystem, CA, USA) with the following cycling conditions:

**First round:** 94°C 1mins, 40cycles of 94°C 30secs, 37°C 30secs, 72°C 90secs, final extension of 72°C for 7mins.

**Second round:** 94°C 1 min, 35 cycles of 94°C 30 secs, 50°C 30 secs, 72°C 90secs, final extension 72°C 7mins. Amplified products were visualized on 2% agarose using Biorad® Image reader.

**Sequence and phylogenetic analysis:** the PCR products were purified and directly sequenced in both directions by a commercial sequencing company (Macrogen®, Korea) using Sanger´s method on ABI 3730XL (Applied Biosystem). Nucleotide sequences were assembled edited and analysed using BioEdit (v7.2.5) and ClustalW in Molecular Evolutionary Genetics Analysis (MEGA) 6.0 [13]. Nucleotide BLAST search on the GenBank data base show homology with rabies virus and sequences with identities to the isolates were retrieved and included in the alignment and phylogenetic tree construction using genotypes 2 and 3 viruses as out groups. To localize the RABV isolate, phylogenetic tree was drawn using Maximum Likelihood method based on the Kimura 2-parameter model in MEGA 6.0 [13]. In addition, evolutionary distances were computed using the Maximum Composite Likelihood Model in MEGA 6.0 [13].

## Results

**Figure 1:**
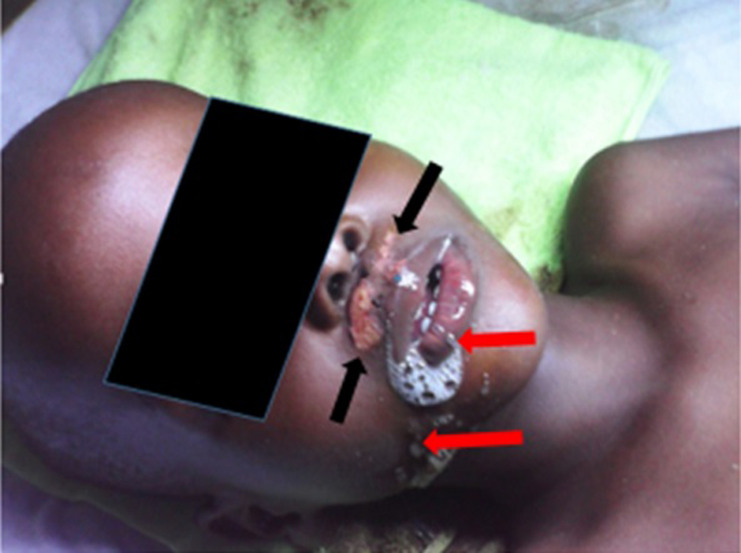
drooling of saliva (red arrows) during clinical stage of infection (2 days before death); Abrasion (black arrows) on upper lip was due to attempts by the patient to remove excessive saliva prior to onset of paralysis

Two days before death of the boy, there was drooling of saliva (Figure 1) in the clinical stage of the infection. The abrasion on his upper lip as seen in Figure 1 was due to his attempts to remove excessive saliva before on set of paralysis. Suckling mice inoculated with saliva specimens remained apparently healthy until signs of sickness were noticed on day 5 post-inoculation (PI) (saliva specimen 4), day 11 PI (saliva specimen 2) and day 12 PI (saliva specimens 1 and 3). On days 12 and 13 PI, some of the inoculated mice became sick with one death recorded while others remained unthrifty till day 15 PI. On day 16 PI, some of the sick mice developed lethargy, with rough coat and were removed on day 17 when they became moribund. After harvesting the moribund mice, the remaining two of the sick ones had in-coordination, lethargy and rough hair coat until day 20 PI when they were found dead.

On the contrary, all the mice inoculated with the CSF remained apparently healthy until day 12 PI. On days 13 and 14 PI, all the mice except one became sick, and from day 15 to 17 were unthrifty. On days 18 and 19 PI, one mouse had posterior paresis, became moribund and was harvested. All the mice with posterior paresis died on day 20 PI. Rabies virus antigen was detected in smears of the brains of the mice inoculated with all the saliva specimens, CSF specimen and the positive control specimen, while there was none in the negative control specimen, when tested by direct fluorescent antibody (DFA).

**Figure 2:**
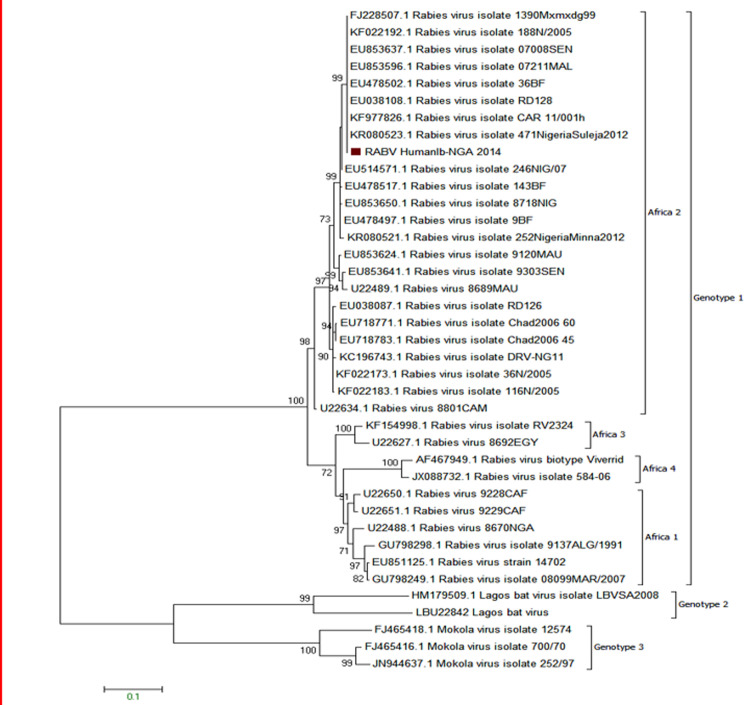
phylogenetic analysis of RABV isolate based on partial nucleoprotein gene from the patient (indicated by red box on the tree) with selected representatives of other genotypes; tree was constructed by the maximum likelihood using MEGA 6.0 with 1000 bootstrap replicates to assign confidence to groupings; the scale bar at the bottom left shows number of substitutions per site

The partial N-gene of the rabies virus from the specimens initially passaged in mice and amplified by RT-PCR produced a band size of 589bp. After sequencing, the contig yielded 1170bp which was trimmed to 628bp of the N-gene coding region. BLAST search of the sequence from the hnRT-PCR product showed relatedness to rabies virus. Phylogenetic analysis of the partial N-gene sequence localized the isolate to Africa 2 viruses of the *Lyssavirus* genotype 1 (classical rabies virus) (Figure 2). Pair-wise nucleotide and deduced amino acid sequence comparison showed 95.7-100% and 94.9-99.5% identity between the isolate and other genotype 1 rabies viruses respectively. In addition, 100% homology was observed with the isolate and rabies viruses from Mali (EU853596), Burkina Faso (EU478502), Senegal (EU853637) and Central African Republic (KF977826).

## Discussion

The 5-year-old boy was bitten by a mongrel in a rural community where human and animal anti-rabies vaccination is hardly carried out, due to poverty and ignorance. The victim was not in position to rationalize or speak intelligibly, but from parental recall it was reported that he started refusing to drink water before admission to tertiary health facility. Hyper-salivation and dyspnoea were evident upon arrival at the referral health facility as depicted in Figure 1. Rabies is a neglected disease that mainly affects the most vulnerable people including children especially in endemic areas [14]. Most human deaths due to rabies occur in resource limited countries particularly in the tropics [15], where the disease is poorly monitored and highly under reported [16]; and where many infected people die at home unreported [3]. In most situations, the incidence of the disease is unknown [17].

Although manifestation of clinical signs and death pattern in mice inoculated with saliva sample and CSF varied, neurological signs of rabies were observed in mice infected with the virus contained in all the specimens, as from day 5 PI. To corroborate this, suckling mice inoculated with each of the samples exhibited unthriftiness and rough coat and RABV antigen was detected in all saliva specimens and CSF by DFA confirmed by RT-PCR (data not shown). This is in contrast to non-detection of rabies antigen from saliva specimen using RT-PCR reported by Jackson *et al*. [18]. Other *Lyssaviruses* namely, Mokola and Duvenhage viruses have caused fatalities in humans in Africa [19,20]. In differentiating between classical rabies and other *Lyssaviruses*, phylogenetic analysis placed this virus isolate in Africa 2 clade and genotype 1, clearly delineating it as classical rabies virus. Using N gene sequence, the topology of phylogenetic tree of our RABV isolate (Figure 2) is in tandem with circulating Africa 2 clade reported previously [21]. Nucleotide analysis showed 95.7% - 100% identity with Africa 2 clade RABVs reported to be endemic in dogs in West and Central Africa [21]. Close genetic relatedness of this RABV isolate with those of Africa 2 clade circulating in West Africa can be attributed to increase cross-border movement of dogs and trading across the sub-region. In some parts of Nigeria and West Africa, dog meat is a delicacy. As such, dog traders are involved in movement of unvaccinated but apparently healthy dogs and dog meat which may be infected with RABV. In a recent study, rabies virus antigen was detected in the brain and saliva of dogs slaughtered for human consumption in south-eastern Nigeria [22].

As observed in this study, management of dog bite cases do not involve first aid treatment or administration of rabies PEP to the victim in many developing countries. This is due to lack of awareness on the danger of rabies, poor knowledge of what to do when human exposure occurs and inaccessibility to human anti-rabies vaccine and RIG, especially in rural communities. This was the case in most Primary Health Centres which are the first point of call when dog bite cases occur. The situation is similar in most secondary and tertiary health centres in Nigeria, where anti-rabies vaccine is not considered as first line treatment in cases of dog bites. In some cases, health workers administer only analgesics, tetanus toxoid and antibiotics to dog bite victims and dress bite wounds. In many instances, bite wounds are even sutured immediately dog bite cases are reported which is contra-indicated in the management of animal bite wounds especially where rabies is suspected [23]. The implication of wound suturing and failure to administer recommended medications is that infection takes its full course or incubation period without interruption and culminates in clinical disease and death. In the present case, appropriate medications were not applied during initial visit to primary health centre, hence the development of rabies in the minor 7 weeks post exposure, which is consistent with its incubation period of 4 - 8 weeks [24].

Treatment of clinical rabies in human is by employing the ‘Wisconsin rabies treatment protocol’ [25]. However, application of this protocol did not succeed in some cases [26]. Karande *et al*. [27] reported rabies in a 6 year old boy bitten by a stray dog in India. The victim received first aid and PEP (including ERIG and 4 out of the 5 doses of Purified Chick Embryo Cell (PCEC) rabies vaccine as recommended by WHO [5] but did not survive the deadly disease. However, in this present case neither first aid nor PEP and rabies immune globulin (RIG) were administered hence the onset of clinical rabies and death which could have been prevented by prompt administration of PEP including RIG before onset of disease [5].

Traditional beliefs and ethical issues relating to obtaining assent of the family of rabies patients for post-mortem sample collection and confirmation have posed major constraints in effective diagnosis of rabies in Nigeria. It is therefore not surprising that 78 human deaths suspected to be rabies-related in 10 states of Nigeria and diagnosed based on clinical manifestations were not confirmed by laboratory techniques [28]. However, Mokola virus infection in human in Nigeria was confirmed by series of laboratory techniques in the last five (5) decades [19]. Some cases of human rabies reported in Europe which were imported from Nigeria were also confirmed by laboratory procedures. Misdiagnosis of rabies as malaria has been identified as a major challenge in assessing its magnitude in parts of Africa [17] where several deaths resulting from the disease go unnoticed and unreported. We, therefore, recommend that rabies should be included in the list of differentials in diagnosis of cerebral malaria and other cases presenting with nervous signs in Nigeria as suggested in Malawi [17]. Although rabies is preventable and a notifiable disease in Nigeria, it has suffered serious neglect due to logistic issues with disease reporting system and limited political will from government authorities. If nothing is done to reverse the situation, it will pose a serious setback in the campaign to put an end to rabies deaths across the world by the year 2030 [29], in Nigeria.

## Conclusion

We confirmed a case of fatal rabies seven weeks after dog bite which occurred in a rural community in south-western Nigeria. This is the first report of laboratory investigation of fatal human rabies transmitted by dog bite in a minor in Nigeria. The classical rabies virus detected belongs to Africa 2 linage commonly found in west and central Africa. The deadly outcome resulted from lack of awareness on the dangers of rabies and the necessary actions to take after exposure and non-availability of rabies PEP in the primary health centre facility where initial report was made. This implies that deaths resulting from rabies occur unreported in rural communities of Africa and underlines the need to educate rural dwellers and health workers on early reporting of incidence of dog bites with tips on first aid and administration of PEP to exposed persons to avoid fatal outcomes. Results of this study support the use of saliva and CSF in *intra vitam* as well as in post mortem diagnosis of rabies when it is not practicable to obtain brain tissue samples.

### What is known about this topic

- Rabies is prevalent in Africa especially, in rural communities and is mainly transmitted by dog bite; with children below age 15 being the most vulnerable;
- In Nigeria, previous studies focused on the ecology, past records of dog and human rabies and diagnosis of animal rabies with little attention given to laboratory confirmation in people with illnesses presenting with symptoms that incriminate rabies;
- Previous studies supported the use of saliva and CSF in intra vitam as well as in post mortem diagnosis of rabies when it is not practicable to obtain brain tissue samples.

### What this study adds

- This is the first report of fatal human rabies transmitted by dog bite in a minor in Nigeria;
- In this study, we describe laboratory investigation of suspected rabies in a 5-year old boy who presented with hydrophobia, photophobia and aerophobia in Nigeria;
- This study confirms the use of saliva and CSF in intra vitam diagnosis of rabies when brain tissue samples are not available for test.
